# Wetting Properties of Transparent Anatase/Rutile Mixed Phase Glancing Angle Magnetron Sputtered Nano-TiO_2_ Films

**DOI:** 10.3390/mi11060616

**Published:** 2020-06-25

**Authors:** Vasiliki Vrakatseli, Ergina Farsari, Dimitrios Mataras

**Affiliations:** Department of Chemical Engineering, University of Patras, GR-26504 Patras, Greece; efarsari@plasmatech.gr (E.F.); dim@plasmatech.gr (D.M.)

**Keywords:** mixed phase TiO_2_, glancing angle, RF magnetron sputtering, reversible wettability

## Abstract

Transparent polycrystalline TiO_2_ thin films have been deposited on unheated glass substrates using RF reactive magnetron sputtering. Depositions were carried out at different glancing angles and with different total gas mixture pressures. The variation of these parameters affected the crystal phase composition and the surface morphology. Depending on the glancing angle and the pressure, rutile, mixed anatase/ rutile and pure anatase were deposited at low substrate temperature. Both hydrophilic and hydrophobic TiO_2_ were obtained, exhibiting fast photoconversion to superhydrophilic upon UV irradiation. The effect of the materials physicochemical properties on the wettability and rate of the UV induced superhydrophilicity is evaluated.

## 1. Introduction

Wetting properties of solid surfaces are of great importance both from a theoretical point of view and for industrial applications. During the last decades, there has been a great research interest focused on the control of the wetting state of real surfaces, towards the fabrication of thin films resembling biosurfaces which exhibit extreme or peculiar wetting characteristics. The well-established studies of Young, Wenzel, and Cassie and Baxter revealed that the wettability of a solid surface (hydrophobic or hydrophilic) is greatly influenced by the intrinsic surface energy and the surface morphology [1,2,3]. Thin films possessing special wetting characteristics may act as self-cleaning surfaces, anti-biofouling surfaces for biomedical applications, on microfluidic devices for micro-droplet manipulation and many more [4,5,6,7,8].

TiO_2_ thin films are considered perfect candidates for all the above mentioned applications, due to their chemical inertness, biocompatibility, photocatalytic properties, and high transparency. Moreover, TiO_2_ films can convert from relatively hydrophilic (50–70°) to superhydrophilic (CA < 10°) by exposure to UV radiation, while the surface gradually regains its original wettability in dark [9]. The prevailing mechanism of the UV-induced hydrophilicity of TiO_2_ suggests that the photogenerated electron-holes migrate to the TiO_2_ surface and induce surface oxygen vacancies which act as energetically favored sites for the dissociative adsorption of water [9,10] The intrinsic wettability in dark, and also the rate of the photoinduced superhydrophilic conversion (PSH) determine the extent of the applicability of the TiO_2_ surfaces. Rough nano/mesoporous surfaces exhibit enhanced hydrophilicity or hydrophobicity and provide large surface area required for the acceleration of the PSH. For this reason, the wetting properties of nanostructured TiO_2_ surfaces have been thoroughly investigated [11,12]. Superhydrophobic TiO_2_ coatings have been prepared by different methods which however usually involve wet chemistry, multistep complex processes, annealing at high temperature, or further physical/chemical surface modification [13,14,15,16,17,18,19]. On the other hand, the deposition of long term hydrophilic TiO_2_ surfaces in the absence of UV exposure, is a more challenging task. In this direction, TiO_2_/SiO_2_ composite films are fabricated, or the hydrophilicity of the TiO_2_ surface is extended by oxygen plasma post treatment [20,21,22,23].

RF magnetron sputtering (MS) is a versatile technique for the deposition of polycrystalline TiO_2_ thin films at low temperature and is also suitable for large scale depositions. At the same time, the glancing angle physical vapor deposition (GLAD) constitutes a common practice towards the fabrication of rough and porous thin films [24]. The combined GLAD magnetron sputtering deposition of TiO_2_ films has been widely studied in terms of the effect of the deposition parameters on the films microstructure. However, there are only a few studies focusing on the TiO_2_ films wettability and PSH conversion, related to the surface morphology and/or structural characteristics induced by the GLAD MS deposition [25,26,27]. In this work, we present the fabrication of TiO_2_ thin films by GLAD RF magnetron sputtering on unheated glass substrates. The effect of the glancing angle and the working pressure on the TiO_2_ films structure and phase composition, the surface morphological characteristics, the optical properties, and the wetting characteristics have been evaluated. Subsequently, the relation between the phase composition and roughness with the wettability of the TiO_2_ films in dark and the photoinduced superhydrophilicity are investigated. 

## 2. Materials and Methods 

### 2.1. Glancing Angle RF Magnetron Sputtering Depositions

**Figure 1:**
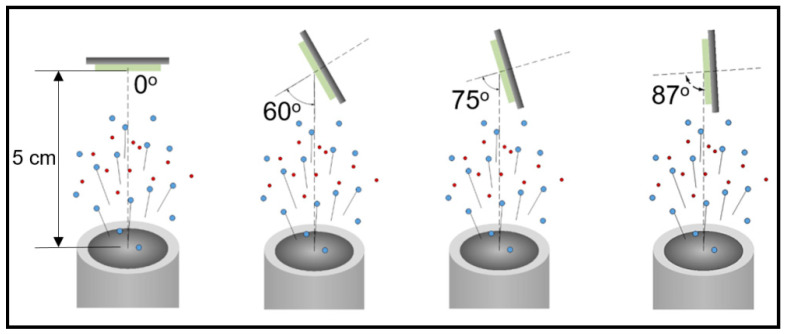
Substrate geometry with respect to target surface for glancing angle depositions.

TiO_2_ thin film depositions on unheated glass substrates were carried out using a custom RF magnetron sputtering system in the reactive mode. The chamber was evacuated to a base pressure of 5 × 10^−7^ Torr by a turbomolecular (Oerlikon SL300, Leybold GmbH, Cologne, Germany) and a dry scroll pump (Edwards XDS10, Edwards Vacuum Inc., NY, USA) in series. A Ti metal target (2 in diameter) attached on balanced magnetrons RF source was sputtered with high purity argon while high purity oxygen was used as the reactive gas. The oxygen content of the mixture was tuned to 50% or 32% in terms of partial pressure. Under these conditions, sputtering is carried out in the oxide mode of the target which favors TiO_2_ stoichiometry. The glass substrates were placed 5 cm above the Ti target at different glancing angles with respect to the target surface (0, 60, 75, and 87°) as illustrated in Figure 1. RF power of 300 W was delivered to the cathode by a RF generator (Cesar, Advanced Energy, Advanced Energy, Fort Collins, CO, USA). The sputtering parameters, as well as the samples nomenclature, are presented in Table 1. 

### 2.2. TiO_2_ Thin Films Characterization

Contact profilometry (Dektak XT, Bruker, MA, USA) was used for the measurements of the film thickness. The TiO_2_ thin films structure was determined by micro-Raman spectroscopy (Horiba Jobin-Yvon LabRam HR800, Horiba, Kyoto, Japan) in the visible range. The surface morphology and surface roughness were obtained by atomic force microscopy (Dimension Fast Scan, Bruker, MA, USA). A Lambda 900 (Bruker) UV–vis–nIR spectrophotometer was used for recording the UV-vis transmittance spectra (300 nm–800 nm) of the deposited TiO_2_ thin films. TiO_2_ thin films wettability was characterized right after the deposition and during prolonged storage of the films in dark ambient conditions. The static contact angle of 2 μL deionized water drops was measured under room temperature and RH 55–60%, using a commercial contact angle goniometer (Kruss, DSA100, Kruss Scientific, Hamburg, Germany). The photoinduced conversion to superhydrophilicity was evaluated by discrete measurements of the sessile drop in time intervals (2–3 min) under exposure in UV radiation [23]. 

## 3. Results and Discussion

### 3.1. TiO_2_ Films Crystal Structure

**Figure 2:**
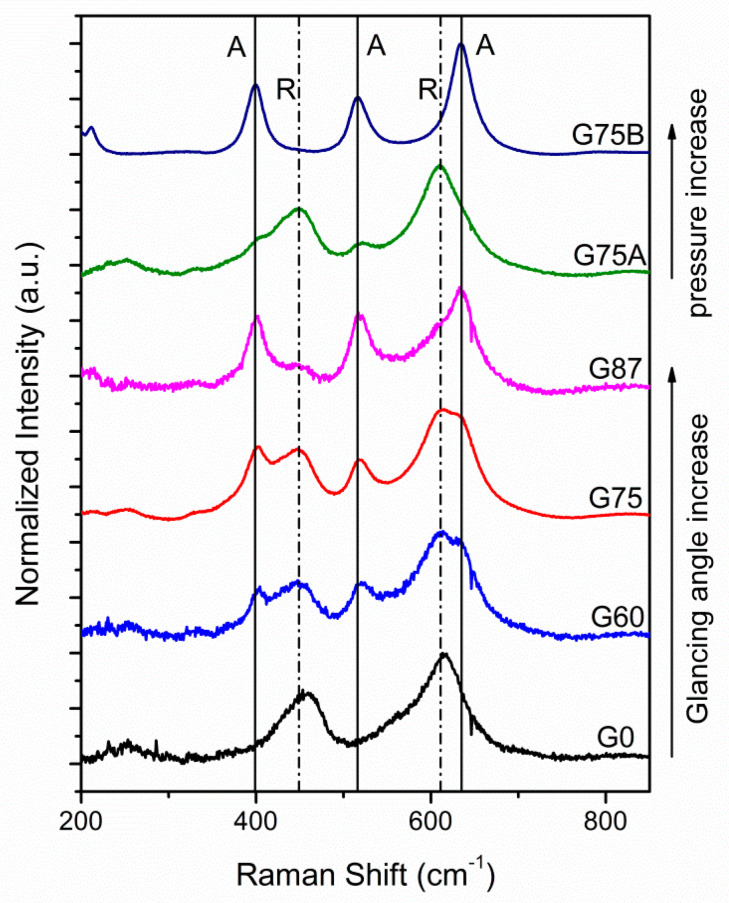
Raman spectra of the TiO_2_ films deposited at different glancing angles and sputtering conditions.

Figure 2 depicts the acquired Raman spectra of the TiO_2_ samples deposited at different glancing angles (0, 60, 75 and 87°) at 2.4 mTorr, and at 75° glancing angle varying the total working pressure (1 mTorr and 4.2 mTorr). Rutile or anatase structure is identified by the Raman active phonon modes in the visible range at 447 cm^−1^ (Ε_g_) and 610 cm^−1^ (A_1g_) and 399 cm^−1^ (B_1g_), 519 cm^−1^ (A_1g_/B_1g_) and 639 cm^−1^ (E_g_) respectively [28,29]. Polycrystalline rutile, mixed anatase/rutile and anatase ΤiO_2_ films were obtained. Phase formation is significantly affected by the glancing angle of the substrate and the working pressure. The increase of glancing angle at constant pressure and the increase of the total working pressure at large glancing angle (75°) resulted in phase shift from rutile to anatase. The effect of glancing angle and pressure on the RF magnetron sputtered TiO_2_ films phase composition has been reported and thoroughly discussed in our recent study [26]. The content of different phases can be estimated by the integrated intensities of the rutile and anatase peaks using the Equations (1) and (2) [30]:

(1) $$\%R=\frac{I_{447}+I_{610}}{I_{399}+I_{447}+I_{519}+I_{610}+I_{639}}$$

and

(2) %A=1−%R

The acquired Raman spectra were deconvoluted and fitted with Lorenzian functions and the calculated values are summarized in Table 2.

### 3.2. Optical Properties

**Figure 3:**
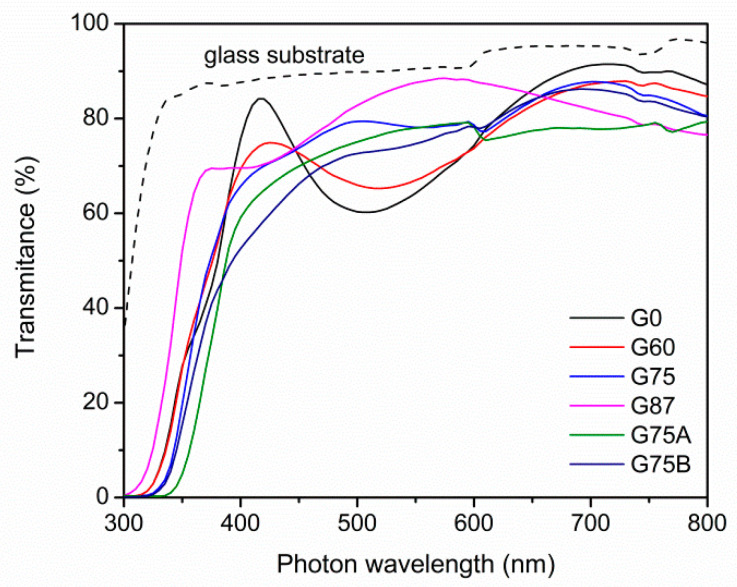
UV-visible optical transmission spectra of the TiO_2_ films on glass substrate.

Figure 3 shows the optical transmittance spectra in the UV-Visible range of the as deposited TiO_2_ thin films on glass substrates. The films were highly transparent in the visible range. The differences in the transparency are attributed to the thickness and the morphology of the films. Among the thinner G0, G60, and G87 (120–170 nm) films, the G87 exhibits the highest transmittance value in the visible range reaching 90%. 

The optical band gap of the indirect transition was evaluated using the well-established Tauc’s relation [31]:

(3) $$\alpha hv=\alpha_{ο}{\left(hv-E_{g}\right)}^{n}$$

where α is the absorption coefficient, $\alpha_{ο}$ is a constant related to the probability of transition, h is Plank’s constant and v is the photon frequency. Depending on the exponent value, the optical band gap, ${Ε}_{g},$ for the allowed direct (n=1/2) or indirect transitions (n=2), can be estimated by plotting the ${\left(\alpha hv\right)}^{\frac{1}{n}}$ vs. hv, the so called Tauc plot. The absorption coefficient is calculated from the transmittance *T* and the thickness d as:

(4) $$\alpha =-\ln\left(T\right)d^{-1}$$

**Figure 4:**
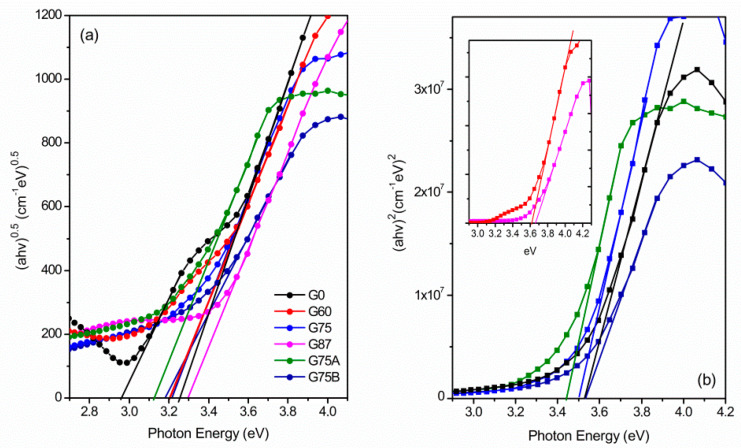
Tauc plots for the estimation of (**a**) indirect band gap and (**b**) direct band gap of the TiO_2_ films.

The Tauc plots showed linear regions of both direct (Figure 4a) and indirect (Figure 4b) allowed transitions of the TiO_2_ films. The corresponding optical Eg values are estimated by the extrapolation of the linear region of the Tauc plots to the x axis.

The calculated band gap values are in the order of 3.1 to 3.3 eV (indirect) and 3.44 to 3.66 (Table 2). The band gap increases with the increase of glancing angle from 0° to 87° or the pressure from 1 mΤorr to 4.2 mTorr, as the phase composition also changes towards anatase. The optical indirect band gap of bulk rutile and anatase is 3.05 eV and 3.2 eV respectively, while larger band gaps are usually reported for the amorphous ΤiO_2_. The nanorutile G0 shows a first indirect transition at 2.95 eV and a second one at 3.25 eV. The first transition is assigned to the rutile crystals while the second one can be attributed to the amorphous phase developed at the early stages of the sample growth. The pure anatase G75B film also exhibits a band gap value very close to that of the bulk phase (3.18 eV). For mixed phase TiO_2_, it has been reported that the proper band alignment of the heterojunctions leads to a red shift of the effective band gap compared the pure bulk phases [32]. For example, band gap of 2.89 eV has been reported for 40% anatase TiO_2_ thin film, which is even smaller than that of pure bulk rutile [33]. However, the mixed phase TiO_2_ films of this study (G60, G75, G87) exhibit wider band gaps compared to the corresponding pure rutile (G0) or anatase (G75B). The enhancement of the band gap, observed in this work, can be attributed to the porosity induced at large glancing angle deposition [34,35]. 

### 3.3. Wettability and Surface Morphology

**Figure 5:**
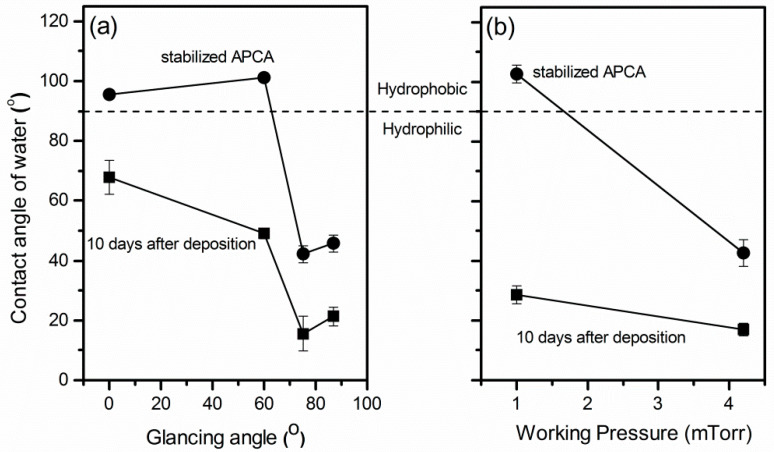
Measured apparent contact angles 10 days after the deposition and after prolonged storage in dark when the surface is stabilized versus (**a**) the glancing angle of the substrate (P_t_ =2.4 mTorr, O_2_ content:50%) and (**b**) the working pressure (glancing angle 75°, O_2_ content: 32%).

Figure 5 shows the water contact angles of the TiO_2_ films as a function of the glancing angle (Figure 5a) and the working pressure (Figure 5b). The increase of the glancing angle and the working pressure apparently affected the wettability of the TiO_2_ films. All the films were hydrophilic after 10 days of storage with apparent contact angles (APCA) ranging from ≈15° to ≈70°, depending on the deposition conditions. Depositions at high glancing angle or pressure resulted in the most hydrophilic surfaces. The films were then left under dark ambient conditions until the contact angle reached a constant value indicating the saturated state of the samples. After prolonged storage (>30 days) the films deposited at small glancing angle or pressure (G0, G60 and G75A) turned to hydrophobic with APCAs > 100°, while those deposited at large glancing angle or high pressure (G87, G75A and G75B) maintained high hydrophilicity with APCAs 40–45°. 

During long term storage the surface energy is inevitably decreased, i.e., the Young contact angle of the TiO_2_ films is increased, either due to the accumulation of non-polar hydrocarbon adsorbates or due to the depletion of polar surface species. However, it is noteworthy that although the conditions and the period of storage were the same for all the films, the augmentation of the APCA, for some of the films was very large, with ΔCA up to 74°, while for others, ΔCA was only 20–25°. The wettability of rough surfaces depends on the Young contact angle, i.e., the contact angle of a corresponding flat surface, the surface roughness and also on the specific topography features of the surface. The implemented deposition parameters caused simultaneous variation of the crystal structure, roughness, and surface morphology of the GLAD TiO_2_ films. Therefore, in order to interpretate the different final wetting states, all the above mentioned factors which affect the final wettability are considered.

**Figure 6:**
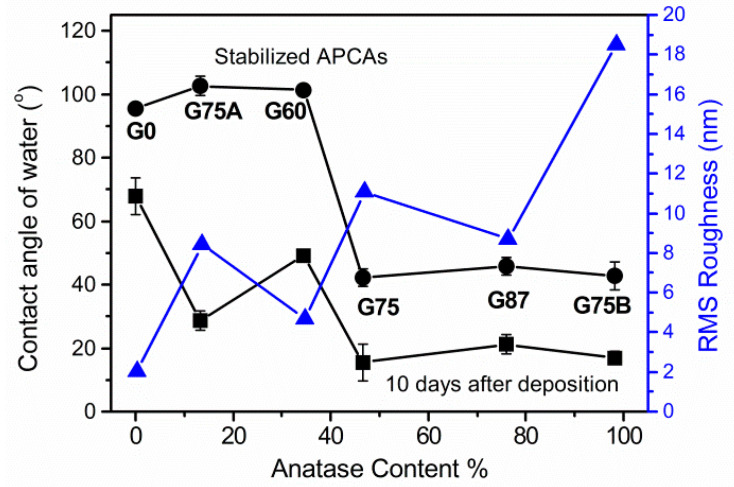
Apparent water contact angles of the GLAD TiO_2_ films, 10 days after the deposition and >30 days after deposition (stabilized), along with the respective RMS surface roughness (in nm), as a function of the TiO_2_ phase composition.

In Figure 6 the APCAs after 10 days of storage and the stabilized APCAs of the TiO_2_ films are plotted against the anatase content. The respective rms roughness values, as they were obtained by 5 μm × 5 μm AFM images, are also shown. The APCAs are well correlated with the content of the anatase phase of the films, regardless of the deposition conditions. It can be seen, that only TiO_2_ films consisted of >45% anatase were highly hydrophilic after 10 days and retained their hydrophilicity. The films that eventually turned from hydrophilic to hydrophobic are the rutile rich films (anatase content < 40%). The effect of the different crystal structure of the surface is more obvious if we compare TiO_2_ films with the same rms roughness (G87 vs. G75A). The contact angle relaxation on polycrystalline mixed or anatase TiO_2_ sputtered films of same roughness in atmospheric conditions was examined by Lee and Park [36] using quantitative XPS studies. It was found that, compared to the films containing rutile, the anatase films retained high hydrophilicity due to the formation of strong donor-acceptor complexes by the interaction of Ti-OH basic groups with H_2_O [36]. Τhis implies that the anatase rich surface can be persistent hydrophilic and inherently more hydrophilic (smaller Young contact angle) compared to the rutile films. However, the large difference of the final APCAs between the rutile rich and anatase rich films (ΔCA > 60°) indicates that the difference of the final wetting states should be further investigated by considering the role of roughness and surface morphology. 

The increase of the glancing angle and pressure resulted not only in the preferential growth of anatase over rutile, but also in a remarkable increase of the TiO_2_ films roughness. Figure 6 shows that as the crystal phase develops towards anatase, the rms roughness is increased from 2 nm to 18.5 nm. However, neither the roughness magnitude alone is an adequate measure for the prediction of the final wetting state of all the TiO_2_ films. For example, the G87 anatase and G75A rutile films were respectively hydrophilic and hydrophobic, despite the fact that the rms roughness value of both was ~8.5 nm, indicating that indeed the crystal phase may have significant impact on the final wetting state. 

The effect of roughness on wettability is usually described by the Wenzel and Cassie-Baxter models [2,3]. In the Wenzel model, R, which is defined as the ratio of the real surface area to the projected (smooth) area, induces a deviation of the apparent contact angle from the Young contact angle as:

(5) $${\;\cos\theta }_{A}={R\;\cos\theta }_{\Upsilon }.$$

**Figure 7:**
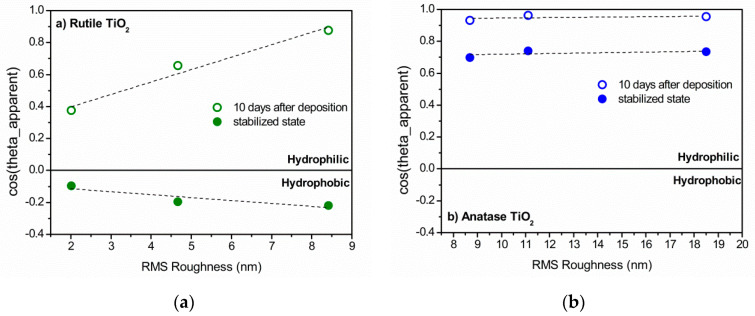
The cosine of the apparent contact angle of glancing angle deposited TiO_2_ films (**a**) rich in rutile (65–100%) and (**b**) rich in anatase (45–100%), as a function of the RMS roughness of the surface.

The Wenzel equation implies that surface wettability, either hydrophilicity or hydrophobicity, is amplified by surface roughness. The Cassie–Baxter model describes the effect of roughness on the apparent contact angle for superhydrophobic surfaces exhibiting low solid-liquid adhesion and low contact angle hysteresis. The latter will not be considered since the water droplet showed strong adhesion on the TiO_2_ films surface and none of the films reached the superhydrophobic state. In Figure 7, the cosine of the apparent contact angle is plotted as a function of roughness for both the rutile and anatase rich films. The rutile rich films follow the Wenzel model. After 10 days of storage, the θ_Y_ of rutile is probably <90° and the increase of roughness induces higher hydrophilicity (cosθ_A_ is increased). A smaller negative slope of cosθ_A_ is also observed for the hydrophobic stabilized state of the rutile films which indicates that after long term storage, the Young contact angle just exceeds 90°. Hence, the transition of rutile TiO_2_ surface from hydrophilic to hydrophobic ΔCA is greater as the surface roughness increases.

On the other hand, the highly hydrophilic character of the anatase rich films 10 days after the deposition (APCAs ≈ 20°) and after its stabilization (APCAs ≈ 40°) cannot be expressed by the Wenzel model. In Figure 7b, cosθ_A_ is shown to be independent of the rms roughness. It is possible that the low apparent contact angles originate from the minimum possible free energy equilibrium of the droplet on the surface among the different wetting states, as described by the Cassie impregnating model (CI) [37,38]:

(6) $${\cos\theta }_{A}=1-f_{s}+f_{s}{\;\cos\theta }_{\Upsilon }$$

In this case, the surface equilibrium is reached by the lateral propagation of the liquid front beyond the droplet, through the surface microstructure. Water penetrates the surface pores and *f_s_* is the fraction of the solid islands of the mixed liquid-solid surface that remain dry. Equation (6) shows that as long as the surface wettability is expressed by the CI model, the APCA is independent of the roughness. The criterion for this wetting mechanism is [37,38]:

(7) $${\cos\theta }_{Y}>\frac{1-f_{s}}{R-f_{s}}$$

or according to the thermodynamic analysis of Huang et al. [39]:

(8) $${\cos\theta }_{Y}>\frac{d}{4h+d}$$

where *d* and *h* are the mean diameter and height of surface pores. Therefore, the thermodynamic equilibrium can be shifted from the Wenzel to the CI state, due to certain texturization and surface porosity. 

**Figure 8:**
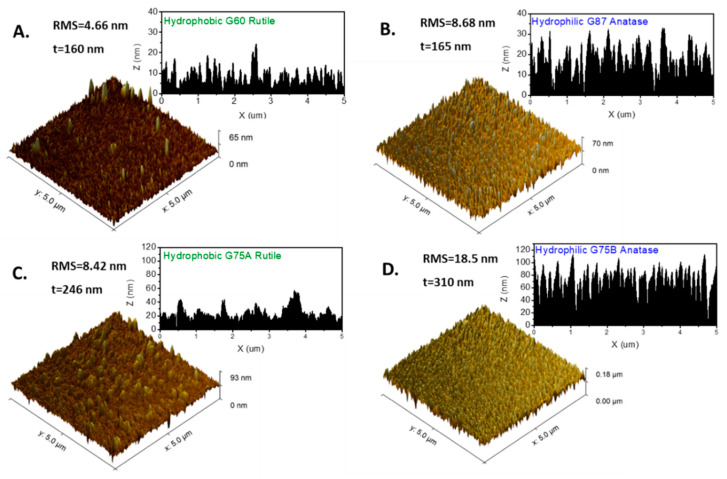
3D views of the AFM images (5 μm × 5 μm) and line height profiles acquired for two hydrophobic and two hydrophilic TiO_2_ thins films. (**A**,**C**): G60 and G75A (hydrophobic rutile), (**B**,**D**): G87 and G75B (hydrophilic anatase)

In Figure 8, we present the AFM 3d images and the cross-section line profiles of two hydrophobic rutile films (A,C) and two hydrophilic anatase films (B,D). Both the hydrophilic anatase films consist of nanorods forming quasi-periodical deep surface pore channels. By the line profiles, the approximate mean height and diameter of the pores for the G87 and G75B anatase films are h = 22 ± 6 nm, d = 150 ± 30 nm, and h = 55 ± 15 nm, d = 140 ± 30 nm respectively. Hence, according to Equation (8), the films are in the CI wetting state if the anatase θ_Υ_ < 65–50°, which is very probable according to ref. [36] as it was previously discussed. The nanorutile films are characterized by irregular needle-like asperities, interrupted by larger structures forming shallow cavities (G60), or mountain-like structures with on-top nanocones (G75A), but no deep pore channels. It is worth noticing that, under the same sputtering conditions, the increase of glancing angle from 60° to 87° (Figure 7A,B), or the increase of pressure from 1 mTorr to 4.2 mTorr (Figure 7C,D), induces prominent changes on the surface texturization, which are related to the phase change from rutile to anatase [26]. 

Subsequently, the wetting state of the TiO_2_ films derives from the synergistic effect of the crystal phase and surface topography. The anatase films probably retain low Young CA and the fibrous structure with deep surface pores facilitates the establishment of the CI wetting state, permitting persistent high hydrophilicity of the surface. In the work of Chaterjee et al. [25], RF sputtered nanostructured TiO_2_ with amorphous structure and shallow pores (<10 nm) exhibited hydrophobicity, despite being deposited at high glancing angle. Therefore, both the anatase structure, as well as the mean pore height, seems to be determining factors for the persistent hydrophilicity of the GLAD TiO_2_ films. On the other hand, the Wenzel state applies for the rutile surfaces with simpler topography. Due to the greater tendency of Young CA to increase during storage, the rutile surfaces exhibit a transition from hydrophilic to hydrophobic, and the ΔCA of this transition depends on the surface roughness. 

### 3.4. UV-Induced Conversion to Superhydrophilic TiO_2_

**Figure 9:**
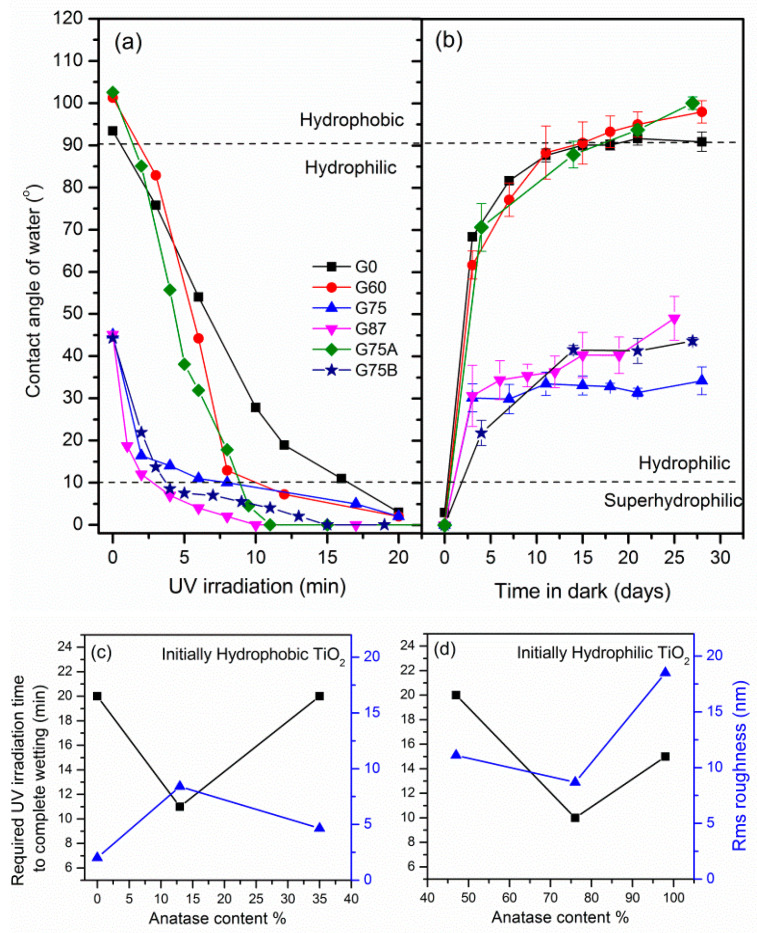
(**a**) Contact angle variation as a function of the elapsed time of exposure of the TiO_2_ films under UV radiation (8 mW/cm^2^) and (**b**) contact angle relaxation during storage of the films in dark ambient conditions after the UV exposure. (**c**,**d**) Required time of UV illumination in order the TiO_2_ films to achieve complete wetting and corresponding surface roughness versus the content in anatase. Note: separate graphs of the initially hydrophobic rutile rich TiO_2_ and the initially hydrophilic anatase rich TiO_2_ are plotted due to the difference in the extent of the superhydrophilic photoconversion. (**c**,**d**) are not comparable.

Figure 9a shows the variation of the water contact angle on the TiO_2_ films as a function of the exposure time to UV radiation. All the films convert to superhydrophilic within some minutes, regardless of the initial hydrophobic or hydrophilic state of the surface, or the crystal phase composition. Interestingly, complete wetting (CA = 0°) occurs within 10–20 min even for the most hydrophobic G75A TiO_2_ film. However, the apparent contact angle of the mixed phase, anatase rich hydrophilic films exponentially decreases with illumination time, while the initially hydrophobic rutile rich films exhibit quasi zero order dependence on the UV exposure time. Therefore, within a very short UV illumination time, the initially hydrophilic high anatase content films reach a critical contact angle, approaching the superhydrophilic condition (CA ≈ 10°), while the corresponding CA of the enhanced rutile hydrophobic films is still substantially large. The different photo-conversion rate order indicates that the high content anatase surfaces become almost saturated by OH groups significantly faster compared to the hydrophobic rutile rich films. Using photoluminescence spectroscopy, Baiju et al. [40] reported that mixed phase anatase-rich TiO_2_ exhibits longer photo-induced carrier lifetime compared to rutile rich mixed phase. Better photoactivity of anatase rich (60–80% A) TiO_2_ materials, compared to pure anatase or rutile rich films has been also reported by several studies and it is attributed to the effective charge separation and transport occurring at the anatase–rutile interface [40,41,42].

Our results are in accordance with those observations. The film deposited at the largest glancing angle G87 (76% anatase) approached the superhydrophilic state within 2 min and required in total 10 min for complete wetting (CA ≈ 0°). The pure anatase G75B and the G75 TiO_2_ film (<50% anatase), converted to superhydrophilic 5 and 10 min slower than the G87 film (76% anatase), despite that their initial contact angle was also ≈45° and that they both exhibited higher surface roughness. Therefore, faster photoconversion of the G87 film is attributed to the crystal phase composition, obtained by the deposition at the largest glancing angle and at P_t_ = 2.4 mTorr. Among the rutile rich TiO_2_ films, the most hydrophobic G75A (13% anatase) and G60 (35% anatase) reach the superhydrophilicity (CA ≈ 10°) in about 8 min, whereas the pure nanorutile TiO_2_ deposited at 0° requires double UV illumination time. Moreover, the G75A deposited at large glancing angle and low pressure, achieved complete wetting very fast, in 11 min. In this case the photoconversion rate of the hydrophobic rutile rich films seems to be better correlated with the films surface roughness rather than the crystal phase composition. These observations are better illustrated in Figure 9c,d where the UV irradiation time required for the TiO_2_ films to attain complete wetting along with the rms roughness are plotted versus the anatase content. Due to the different initial wetting state of the rutile or anatase rich films, two separate graphs are presented. 

After the UV-induced superhydrophilic conversion, all the films were stored in dark and ambient conditions. The recovery of the surface was recorded by contact angle measurements for 30 days (Figure 9b). The TiO_2_ surfaces are gradually reconstructed back to their initial state due to the substitution of the metastable hydroxyl groups by ambient oxygen. Full recovery of the surface is only attained after 25 days for most of the films. Nevertheless, the rate of contact angle relaxation in dark could be enhanced by the application of mechanochemical methods [43].

## 4. Conclusions

Polycrystalline TiO_2_ thin films were deposited using RF reactive magnetron sputtering in glancing angle configuration on unheated substrates. Depending on the glancing angle, or the working pressure the films exhibited different phase composition. The increase of glancing angle or pressure resulted in phase shift from rutile to anatase and in roughness enhancement. All films were transparent in the visible range and the indirect band gaps were estimated from 2.95 tο 3.3 eV. By altering the glancing angle or pressure the realization of either hydrophobic or persistent hydrophilic TiO_2_ films was achieved. After long term storage in dark environment, the TiO_2_ films deposited at low glancing angle or pressure turned to hydrophobic, while those deposited at high glancing angle or pressure showed permanent hydrophilicity. It was revealed that the final wettability, as well as the surface roughness of the nanostructured TiO_2_ films is well correlated with the crystal phase composition. Permanent hydrophilicity is achieved for the films which are rich in anatase and exhibit high surface roughness, while the rutile rich films with lower mean roughness became hydrophobic. Regardless of the rms roughness value, the hydrophilic anatase films deposited at large glancing angle exhibited completely different surface topography features, compared to those of the rutile rich film. By considering fundamental wetting models, the mechanism of different wetting was identified. Permanent hydrophilicity of the anatase films was associated to the Cassie impregnating state, the establishment of which is facilitated by the deep surface pores formed between the anatase nanorods, while the simpler topography rutile rich films were found to follow the Wenzel model. Both the intrinsic wettability and the crystal phase formed under different glancing angles or pressure seem to be crucial for the final wetting state. Regardless of the initial wettability, all the glancing angle deposited TiO_2_ films showed fast photoinduced superhydrophilic conversion with exposure to UV irradiation for a few minutes.

## Figures and Tables

**Table 1:** Samples nomenclature and deposition parameters.

Sample Coding	Glancing Angle (Degrees)	RF Power (Watts)	Working Pressure P_t_ (mTorr)	Oxygen Content (%)
G0, G60, G75, G87	0, 60, 75, 87	300	2.4	50%
G75A, G75B	75	300	A:1, B:4.2	32%

**Table 2:** TiO_2_ thin films thickness, phase composition, % transmittance in the visible range on glass substrate and indirect/direct band gap.

Sample	Thickness (nm)	Anatase Content (%)	Average Transmittance (Vis) %	Direct Allowed Transition Eg (eV)	Indirect Allowed Transition Eg (eV)
G0	140 ± 35	0	74.3 ± 10.4	3.53	2.95, 3.25
G60	160 ± 21	35 ± 5	73.8 ± 6.9	3.61	3.2
G75	240 ± 11	47 ± 3	78.5 ± 5.1	3.5	3.23
G87	165 ± 25	76 ± 6	82.2 ± 6.1	3.66	3.3
G75A	246 ± 29	13 ± 3	74.6 ± 5.0	3.44	3.13
G75Β	310 ± 22	98 ± 1	74.3 ± 8.8	3.53	3.18
